# A Protocol for AI-Powered Tools to Enhance Mobility and Function in Older Adults: An Evidence and Gap Map

**DOI:** 10.3390/jpm15010029

**Published:** 2025-01-14

**Authors:** Mirella Veras, Jordi Pardo, Mê-Linh Lê, Cindy Jussup, José Carlos Tatmatsu-Rocha, Vivian Welch

**Affiliations:** 1 Department of Physical Therapy, College of Rehabilitation Sciences, University of Manitoba, Winnipeg, MB R3E 0T6, Canada; 2 Centre on Aging, University of Manitoba, Winnipeg, MB R3T 2N2, Canada; 3 Ottawa Centre for Health Equity, Faculty of Medicine, University of Ottawa, Ottawa, ON K1H 8M5, Canada; 4 College of Pharmacy, University of Manitoba, Winnipeg, MB R3E 0T5, Canada; 5 Neil John Maclean Health Sciences Library, University of Manitoba, Winnipeg, MB R3E 0J9, Canada; 6 Patient Partner, Bedford, NS B4A 0H6, Canada; 7 College of Medicine, Postgraduate Program in Physiotherapy and Functionality, Federal University of Ceará-UFC, Fortaleza 60430-160, Ceará, Brazil; 8 Bruyère Research Institute, University of Ottawa, Ottawa, ON K1N 5C7, Canada; 9 School of Epidemiology and Public Health, University of Ottawa, Ottawa, ON K1H 8M5, Canada

**Keywords:** evidence gap map, older adults, digital health, equity, artificial intelligence, mobility, function and rehabilitation

## Abstract

**Introduction**: Artificial intelligence (AI) is transforming healthcare by enhancing diagnostic accuracy, treatment, and patient monitoring, benefiting older adults by offering personalized care plans. AI-powered tools help manage chronic conditions and maintain independence, making them a valuable asset in addressing aging challenges. **Objectives**: The objectives are as follows: 1. To identify and describe AI-power-based exercise programs for older adults. 2. To highlight primary evidence gaps in AI interventions for functional improvement and mobility. 3. To evaluate the quality of existing reviews on this topic. **Methods**: The evidence gap map (EGM) will follow the five-step method, adhering to the Campbell Collaboration guidelines and, if available at the time of reporting, PRISMA-AI standards. Guided by the Metaverse Equitable Rehabilitation Therapy framework, this study will categorize findings across domains like equity, health service integration, interoperability, governance, and humanization. The study will include systematic reviews, randomized controlled trials, and pre-and post-intervention designs. **Results** will be reported following PRISMA-AI guidelines. We will use AMSTAR-2 Checklist for Analyzing Systematic Reviews on AI Interventions for Improving mobility and function in Older Adults to evaluate the reliability of systematic reviews and focus on internal validity. **Conclusions**: This comprehensive analysis will act as a critical resource for guiding future research, refining clinical interventions, and influencing policy decisions to enhance AI-driven solutions for aging populations. The EGM aims to bridge existing evidence gaps, fostering a more informed, equitable, and effective approach to AI solutions for older adults.

## 1. Introduction

Artificial intelligence (AI) is changing the healthcare landscape, redefining how health professionals diagnose, treat, and monitor patients [1]. Through the integration of AI technologies, healthcare providers are gaining tools to enhance the quality of care [2]. AI’s impact extends to various aspects of healthcare and provides possibilities for mitigating human errors and enhancing clinical outcomes [1]. It also offers ways to improve function, mobility, and overall well-being [1,3]. Additionally, AI facilitates better health monitoring by analyzing vast patient data, allowing for the early detection of potential issues and tailored interventions [1]. The result is a paradigm shift towards personalized care and independent living, where patients receive treatments and support tailored precisely to their unique needs, ultimately improving overall healthcare outcomes and patient experiences.

As the aging population continues to grow, ensuring access to high-quality care is becoming increasingly important in supporting the independence and quality of life of older adults. While AI holds promise as one of many tools that can aid in this process, it is still one of several factors contributing to this goal. Population aging is a global issue of increasing concern, with projections indicating that the number of individuals aged 65 and older will be approximately 1.5 billion by the year 2050 [3]. This demographic shift highlights the pressing demand for innovative approaches that empower older adults to maintain their independence and foster healthy aging. Older adults are at a higher risk of developing chronic conditions [4]. Regular physical activity and rehabilitation exercises are essential for preventing and managing these health issues effectively.

AI emerges as a potent tool for addressing the complex challenges associated with aging [3]. AI-powered systems offer tailored solutions for older adults, ranging from medication management, smart homes, and fall detection to navigation, thereby creating a safer environment that contributes to their enhanced ability to live independently with autonomy as long as possible [3,5]. AI can assist individuals with physical activity guidelines and personalized care plans [6]. AI-powered assistants can plan, design, and track the physical activities of older adults with chronic diseases, ensuring they stay active and manage their conditions effectively [7]. These AI solutions provide customized exercise recommendations based on individual needs and abilities, offering real-time feedback and adjustments to optimize their fitness routines [7]. AI’s potential as a nursing care support tool is particularly promising as it can create robotic technology to alleviate the workload of caregiving staff in long-term care facilities [3,5].

With the widespread dissemination and growing adoption of AI in healthcare, it becomes imperative to systematically map the evidence surrounding the utilization of AI to enhance the function and mobility of older adults. The significance of this evidence map is twofold: firstly, it serves as a comprehensive summary of the existing efforts in this domain, shedding light on the progress made thus far. Secondly, it plays a role in informing future research directions, ensuring that advancements in AI continue to benefit and improve older adults’ health outcomes.

## 2. Objectives

The objectives of the study are as follows:

1. To identify and extract information from relevant studies on AI-power-based exercise programs aimed at enhancing mobility and functional in older adults: this review will focus on any settings, excluding hospital settings.
2. To identify key areas where there is a lack of primary evidence on the effectiveness of AI interventions for improving function and mobility in older adults, specifically where few or no studies have been conducted to assess their impact.
3. To summarize the quality of the reviews by assessing their methodology, rigor, and relevance to the field.
4. To describe the tools, devices, and resources within the domain of AI that are employed to enhance the mobility and function of older adults while highlighting their functionalities.

## 3. Materials and Methods

Evidence synthesis methodologies are evolving to meet different objectives, with new variations emerging alongside traditional methods like systematic reviews and meta-analyses [8]. While these classical methods provide an understanding of specific questions, they are resource-intensive and limited in scope [9]. To address a variety of needs, alternatives have been developed, such as rapid reviews for urgent deadlines, scoping reviews for broader literature without detailed synthesis, and realist reviews that focus on complex interventions [9,10]. These new methods are still developing, and defining their unique contributions remains challenging. Standardized processes exist for systematic reviews and meta-analyses, but for methods like rapid reviews, realist reviews, and scoping reviews, official guidelines are still emerging [9,10].

Evidence mapping, the latest addition to these synthesis methods, is still in the early stages of development and has not yet been subject to the same level of examination as other methodologies [9]. Although both scoping reviews and evidence maps aim to map the literature, they differ in their approach—scoping reviews provide descriptive summaries while evidence maps highlight gaps in the evidence [11]. There is still debate over the methodology and reporting standards for evidence maps as various definitions and classifications exist. With the increasing number of evidence maps published, there is a need to assess their commonalities and differences and the potential for standardizing methods and reporting in future work [9].

An evidence gap map (EGM) is a relatively recent type of review that organizes and presents the existing evidence in a specific research area [9]. Unlike traditional reviews that summarize the findings of the evidence, EGMs focus on cataloging the available evidence itself [12]. The main objectives of an EGM are to help researchers and practitioners navigate the existing evidence and identify areas where there is a lack of high-quality data [12,13]. Additionally, EGMs highlight evidence gaps that need to be addressed through further synthesis, such as through systematic reviews or by conducting more primary research [12].

EGM method was selected for this review because it effectively organizes and identifies available evidence, especially in emerging areas like AI-powered exercise programs for older adults. The review’s first objective is to extract relevant information from studies on AI interventions aimed at enhancing mobility and function in older adults, excluding hospital settings. EGM is ideal for mapping existing studies and providing a clear overview of the evidence. It will also help identify gaps in the effectiveness of AI interventions, highlighting areas with limited research. Additionally, EGM will assess the quality of reviews by evaluating their methodology and rigor. Lastly, this map will describe the tools, devices, and resources used in AI interventions to enhance mobility and function. Overall, EGM method offers a structured approach to achieving these objectives and provides a comprehensive framework for mapping the evidence.

**Figure 1:**
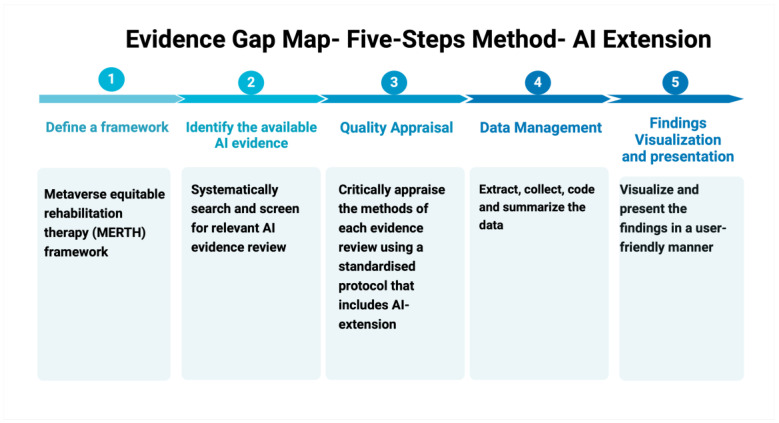
Evidence-gap-map 5-step method with AI Extension for AI-powered tools to enhance mobility and function in older adults.

This evidence-gap-map five-step method with AI Extension was adapted from the five-step method proposed by Welch et al., 2021 [14], which had been adapted from other researchers (Bragge et al., 2011 [15]; Lum, Koper Christopher, & Telep Cody, 2011 [16]; Snilstveit et al., 2013 [17], 2016 [18]) (Figure 1). This EGM also follows the guidance for producing a Campbell evidence and gap map [13]. The results will be reported using PRISMA or PRISMA-AI guidelines if available at the time of reporting [19]. This study is registered with PROSPERO (registration number: 603666).

### 3.1. Framework

**Figure 2:**
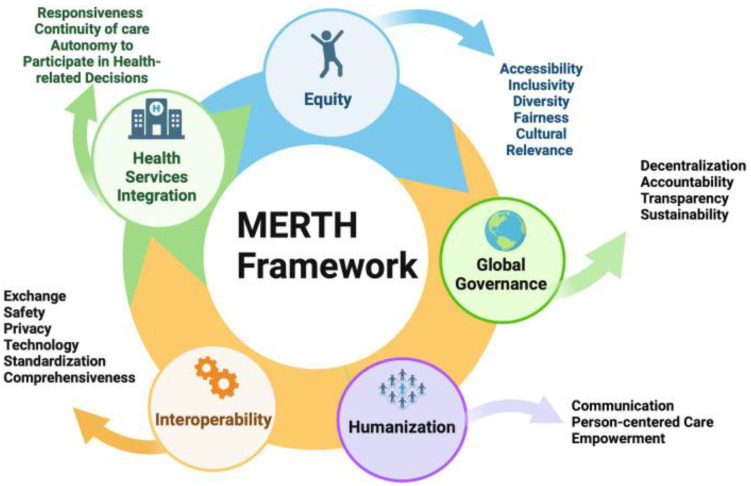
Metaverse Equitable Rehabilitation Therapy (MERTH) framework [21].

Two frameworks have been employed in this protocol and will be used in the study: Population, Intervention, Comparator, Outcomes, Timing, Setting, and Study Design (PICOTSS) [20] and Metaverse Equitable Rehabilitation Therapy (MERTH) (Figure 2) [21] frameworks, each serving distinct purposes. The PICOTSS framework guides the formulation of search terms and the organization of the literature review, ensuring a systematic and comprehensive search process. In contrast, the MERTH framework focuses specifically on equity considerations within the study. It is used to evaluate disparities in access to and outcomes from AI-powered interventions, emphasizing demographic variables such as gender, socioeconomic status, and geographic location. The integration of these frameworks ensures methodological rigor in evidence synthesis while providing a comprehensive analysis of equity issues, supporting the study’s broader objectives of promoting inclusivity and fairness in AI technologies for older adults.

The MERTH framework consists of five key elements designed to guide the integration of rehabilitation interventions, with a focus on ensuring equity, humanization, interoperability, global governance, accessibility, and health service integration. These elements will be adapted to the context of AI-driven rehabilitation therapy to address the unique challenges and opportunities presented by AI technologies [21]. The five elements are described below:

1. Equity: This element focuses on accessibility, inclusivity, diversity, fairness, and cultural relevance [21]. It ensures that AI-driven rehabilitation services are available to all individuals, particularly racialized or underserved populations, while being sensitive to their cultural and socioeconomic contexts.
2. Health Service Integration: This includes responsiveness, continuity of care, and the autonomy [21] to participate in health-related decisions. It ensures that AI-driven rehabilitation services are integrated across various levels and settings, offering a cohesive experience for patients and empowering them to be active participants in their care.
3. Interoperability: This element addresses exchange, safety, privacy, technology standardization, and comprehensiveness [21]. It ensures that different AI-driven healthcare systems and platforms can be integrated, facilitating secure and efficient data exchange and service delivery, while upholding privacy and safety standards.
4. Global Governance: Focused on decentralization, accountability, transparency, and sustainability [21], this element explores how AI-driven healthcare systems can operate in a globally equitable and inclusive manner. It emphasizes the importance of collaborative decision-making, adherence to international regulations, and ethical practices, ensuring that governance structures are transparent and accountable and promote fair access to AI-powered rehabilitation services worldwide.
5. Humanization: This element centers on communication, person-centered care, and empowerment [21]. It ensures that AI-driven rehabilitation interventions maintain the human touch by fostering strong communication between providers and patients while emphasizing personalized care that empowers individuals to take control of their health and well-being. The goal is to leverage AI technology in a way that enhances older adults’ experiences, ensuring empathy, respect, and support in all interactions.

For this study, the MERTH framework has been adapted to guide the development of AI-driven rehabilitation interventions for older adults. The framework’s comprehensive approach will ensure that these interventions are technologically advanced, equitable, safe, and responsive to the diverse needs of older adults. Incorporating these five key elements, the MERTH framework will serve as a critical guide to analyze whether AI-driven rehabilitation therapies are inclusive, effective, and centered on the well-being of older adults.

The MERTH framework will be used to code and categorize our findings systematically, ensuring a thorough and structured approach to identifying and addressing these key areas. Within this framework, five essential domains emerge: equity, health service integration, interoperability, global governance, and humanization through the application of artificial intelligence. This framework will serve as our guide to map the critical gaps and challenges in these fields of AI.

### 3.2. Advisory Committee

The Advisory Committee, an interdisciplinary group comprising clinicians, academics, older adults, AI ethics experts, industry representatives, and professionals from fields such as physiotherapy, occupational therapy, and nursing, will play a pivotal role throughout this study. It will also include individuals with lived experiences, such as a user with firsthand experience, an older adult, and a member of an older adult’s family, ensuring the project remains inclusive and user-focused. To further promote equity, diversity, and inclusion (EDI), the committee will include racialized members from clinical, academic, decision-making, and user backgrounds. The committee will provide guidance at key stages of the study, including protocol development, feedback on the data extraction tool, data analysis, and the creation of the EGM, advising on important visualizations and priorities. Meetings will occur at different phases of the study, such as during protocol and tool development and data analysis, with additional sessions as needed, using methods like virtual or in-person meetings, document reviews, and collaborative workshops. This structured approach will ensure that the Advisory Committee’s expertise and diverse perspectives are integral to shaping the study’s design, execution, and outcomes.

At this stage of protocol development, recruitment for the Advisory Committee is ongoing. Participation has already been confirmed from users with lived experience, clinicians, researchers, and AI ethics experts. Additional users and members with diverse expertise will continue to be recruited to further enrich the committee’s perspectives. In line with a commitment to equity and accessibility, priority will be given to individuals and communities facing systemic barriers, including those related to digital health. This approach will ensure that the committee reflects a wide range of experiences and fosters inclusive and equitable outcomes.

### 3.3. Dimensions

#### 3.3.1. Type of Study Design

This study will include a wide range of studies to provide a comprehensive overview of AI-powered exercise interventions. The included study designs will include the following:

Systematic Reviews: To synthesize existing evidence on AI interventions.

Randomized Controlled Trials (RCTs): To assess the efficacy and effectiveness of AI-based exercise programs.

Quasi-experimental Studies: This category will include studies where participants were allocated through alternate assignment or exogenous variation in treatment allocation (natural experiments) or where self-selection occurred through investigators or participants. Studies using non-equivalent comparison groups (i.e., a comparison group for the intervention) or pre–post analysis will also be considered.

Dissertations and conference abstracts will be excluded from the analysis.

#### 3.3.2. Population (P)

The EGM-AI study will include individuals aged 60 years and older, aligning with the United Nations’ designation for older adults. The United Nations (UN) has established the age of 60 and above as the threshold for categorizing an individual as an “older person.” This criterion expands the scope of eligibility for projects aimed at addressing aging-related development as recognized by the UN [16].

#### 3.3.3. Intervention (I)

The interventions will be categorized into the following areas: Exercise and Rehabilitation Programs, Functional Assessment Tools, Gamification and Motivation Tools, Educational and Guidance Platforms, and Social Interaction and Support Networks (Table 1).

#### 3.3.4. Comparator (C)

The comparators will include the following categories: Traditional Exercise Programs, Non-AI-Based Digital Tools, Fitness Coaching or Rehabilitation, No Intervention (Control Group)/Waitlist, and Usual Care (Table 2).

#### 3.3.5. Outcome (O)

The outcomes will be categorized into the following areas: Physical Function and Mobility, Patient-Reported Outcomes, Clinical/Safety Outcomes, and Engagement and Usability (Table 3).

### 3.4. Data Management

#### 3.4.1. Literature Search

A librarian, in collaboration with research team members, will conduct the search, and the formulation of all search terms will be grounded in the Population, Intervention, Comparator, Outcomes, Timing, Setting, and Study Design (PICOTSS) framework [20]. Search terms will include both database-specific controlled vocabulary as well as keyword searching. The search will undergo peer review [26] before being run in Ovid MEDLINE. It will be translated and run in Embase (Ovid), Cochrane Library, CINAHL (EBSCOhost), Scopus, PEDro, and IEEE Xplore. No language or study type restrictions will be used, but results will be limited to the last ten years due to rapid changes in artificial intelligence capabilities. All results will be uploaded into a platform for deduplication and screening.

#### 3.4.2. Screening and Study Selection

Two reviewers will conduct an independent screening of article titles and abstracts retrieved from the search. The screening process will be based on study criteria. For potentially eligible studies, full-text articles will be retrieved and subjected to further evaluation. Any discrepancies or conflicts in the screening results will be resolved through discussion between the two reviewers or, if necessary, by involving a third reviewer.

#### 3.4.3. Data Extraction

Two reviewers will work independently to extract data from the included published studies. Our data extraction process will be guided by the coding categories outlined in our frameworks. Screening, full-text review, data extraction, and quality assessment will be conducted utilizing the web-based data management platform Covidence [27] in conjunction with EndNote X9.3.3 (Clarivate) and EPPI-Reviewer [28]. The first reviewer will revise the set of included studies and extract data on publication year, gender, age, ethnicity, settings, geographical locations of the studies, AI tool type and descriptor, and research design into a standardized format. Throughout the review process, considerations regarding equity, ethics, safety, confidentiality, and privacy will be assessed, particularly in relation to the use of AI tools and the management of sensitive data. A second reviewer will conduct random checks on the data extraction process, and a third reviewer will be consulted to resolve any disagreements between the primary reviewers. The reference list will be first imported into Covidence, and after the screenings, the included studies will be imported to EPPI-Reviewer 6, developed by the EPPI-Centre (Evidence for Policy and Practice Information and Co-ordinating Centre) at the Social Science Research Unit, University College London (UCL) Institute of Education, UK, is the software used for this study. where predefined coding for the key domains and filters will be applied to each record by one reviewer. A second reviewer will randomly check the coding process, and a third reviewer will be consulted to address any discrepancies. Additionally, the review will examine equity disparities in access and outcomes to ensure that findings are relevant in terms of equity for diverse populations. 

## 4. Synthesis of Results

The data analysis process will be systematically aligned with the objectives and methodologies outlined in our research framework. The coding categories for the data synthesis will follow the key elements of the MERTH framework as described in the methods section. The MERTH framework consists of five core elements designed to guide the integration of AI interventions as described previously. Aligning our coding process with this framework will ensure a systematic categorization of data on study design, population characteristics, intervention types, outcomes, and methodological quality. This approach will ensure that the analysis remains comprehensive, equitable, and focused on addressing the needs of diverse older adults’ populations, ultimately highlighting knowledge gaps and informing future research priorities.

Upon completing the data extraction phase, we will meticulously organize and categorize the collected information. This will involve synthesizing data while adhering to predefined coding categories and criteria. We will employ various analytical techniques, such as quantitative analysis to identify gaps in the existing literature, qualitative analysis to assess the quality and relevance of available evidence, and thematic analysis to uncover emerging patterns and trends within the identified research landscape.

Quantitative data will be succinctly summarized using descriptive statistics. Our reporting will include the presentation of counts and percentages, accompanied by 95% confidence intervals (CIs) for medians and categorical variables. Furthermore, for continuous variables, we will provide interquartile ranges as part of our summary.

Additionally, our data analysis will prioritize transparency and rigor, and any discrepancies or uncertainties will be systematically addressed through consensus among our research team members. Ultimately, this data analysis process will provide a comprehensive overview of existing knowledge gaps in our chosen field, enabling us to identify priority areas for further research and evidence synthesis.

### 4.1. Analysis of Subgroups

In this EGM, we will investigate specific subgroups based on population characteristics, intervention types, and equity aspects. We will further categorize the participants based on the types of AI-powered interventions they receive, which include exercise and rehabilitation programs, functional assessment tools, gamification and motivation tools, educational platforms, and social interaction networks.

Additionally, we will assess subgroups by comparing different comparators such as traditional exercise programs, non-AI-based digital tools, and usual care. We will also consider equity aspects by examining demographic variables such as gender, socioeconomic status, and geographic location to identify disparities in access to and outcomes from AI-powered interventions. The planned analytical approach will involve stratifying the data according to these subgroup definitions, allowing us to explore variations in outcomes such as physical function, mobility, user engagement, and overall health equity. This sub analysis will aim to identify trends and differences among the various intervention types and participant characteristics, providing a comprehensive understanding of how AI tools impact mobility and functional capabilities in older adults, with particular attention paid to equity considerations as recommended by the MERTH framework [21].

The definitions of subgroups for this analysis will be based on the key population, intervention, and equity characteristics as outlined in the methods section. Specifically, subgroups will be defined by population characteristics such as age, gender, and socioeconomic status, as well as by intervention types including AI-powered exercise, functional assessment tools, gamification and motivation tools, educational platforms, and social interaction networks. Equity-related subgroups will be defined using demographic variables such as gender, socioeconomic status, and geographic location to examine disparities in access and outcomes. These subgroup definitions will guide stratification and analysis, ensuring an understanding of the data and alignment with the MERTH framework’s emphasis on equity and accessibility.

### 4.2. Risk of Bias

An adapted standardized checklist, AMSTAR-2 [29] (Appendix B), will be used to assess the confidence in the conclusions drawn from each systematic review, following the recommendations of Saran and White (2018) [30]. In the context of evidence gap maps, only the risk of bias in the systematic reviews will be assessed, rather than in individual studies. This approach is informed by the complexities associated with evaluating the external validity of individual studies, which relates to the generalizability of research findings. Given the broad and diverse topics typically included in systematic maps, focusing our appraisal on the systematic reviews will allow us to more effectively gauge the reliability and quality of the overall evidence [26]. The rationale behind this approach stems from the inherent complexities associated with assessing external validity, which pertains to the generalizability of research findings, especially given the broad and diverse topics typically included in systematic maps [14].

## 5. Discussion

This study, centered on the evidence and gap map of artificial intelligence in the context of tools designed to enhance mobility and function in older adults, will aim to shed light on how AI can support exercise monitoring, remote monitoring, and mobility improvement. The power of these tools to enable remote monitoring will also be explored, including in rural and Indigenous communities, where access to healthcare may be limited. The findings will highlight promising applications of AI while identifying critical research gaps requiring further exploration. To facilitate understanding and interpretation, the evidence and gap map will be presented in a visually accessible format.

### 5.1. Visualization Analysis and Presentation

A descriptive synthesis of the evidence related to AI tool types and descriptors will be developed, highlighting the year-wise and geographical distribution of the included studies based on reported affiliations, with particular attention given to systematic reviews. The coded studies will be plotted in EPPI-Reviewer to create a visual evidence map. Studies will be plotted in multiple locations on the evidence gap map if they have been cited more than once across different research sections. The resulting EGM will visually depict the number of cited studies through the size of the spheres, utilizing a color-coding system to differentiate between various categories of AI-based intervention programs designed to improve mobility and function in older adults. Specifically, red will represent low-frequency categories such as rarely cited applications like robotic-assisted rehabilitation programs. Orange will indicate moderate-frequency categories including commonly referenced AI tools like wearable devices that monitor physical activity or machine learning algorithms used in gait analysis. Green will denote high-frequency categories that encompass widely studied AI-based programs such as AI-driven telerehabilitation platforms and interactive mobile applications that have demonstrated effectiveness in enhancing mobility and functionality. Blue will be used for unclassifiable categories that do not fit into the defined frequency levels. This approach will help highlight areas with significant research activity, as well as those that may require further investigation. Additionally, the final report will include a section discussing the overall status of research on AI tools within the context of the study, emphasizing gaps, under researched categories, and areas with low-level evidence. Final tables and figures will also feature a PRISMA diagram and visualizations depicting the geographical and AI-tool distributions of the selected studies.

This visualization will also include the following:

- A clear categorization of AI technologies by their impact areas (e.g., mobility and function enhancement).
- The identification of well-researched areas versus those with limited evidence.
- Graphical representations such as heat maps, charts, and infographics to illustrate the distribution of evidence and gaps.

The dissemination of findings will target a broad audience, including clinicians, researchers, users, decision-makers, technology industry developers, and funding agencies, with a focus on advancing equity, accessibility, and innovation in AI-powered technologies for older adults. The goals will be as follows:

To Guide Researchers and Funding Agencies: It will highlight priority areas for future research and funding to ensure the development of equitable and accessible AI technologies that maximize their impact on older adults’ health, mobility, and safety through exercise and physical activity. Funding agencies can use this study to identify evidence gaps and strategically allocate resources toward research that addresses these gaps, prioritizes underserved populations, and advances the development of inclusive and impactful AI solutions.

*To Bridge Healthcare Gaps:* The study will empower healthcare providers to address existing gaps in services, particularly for underserved populations, and improve health outcomes for older adults through accessible and inclusive rehabilitation interventions. Clinicians can leverage these AI-powered tools to advance equity by delivering personalized and remote care, especially in rural, Indigenous, and other underserved communities, where access to healthcare services is limited. This approach ensures that older adults, regardless of their geographic locations or socioeconomic statuses, have equitable access to tools that enhance mobility, function, and overall well-being.

*To Promote an Equity Lens:* It will inform the development of AI-driven tools with an emphasis on equity, ensuring that technologies are inclusive, accessible, and responsive to the needs of diverse populations, including rural and Indigenous communities. Prioritizing equity and accessibility, this EGM will seek to drive the development of technologies that improve function, mobility, and overall quality of life for all older adults. This visualization will also include a clear categorization of AI technologies by their impact areas (e.g., mobility and function enhancement), the identification of well-researched areas versus those with limited evidence, and graphical representations such as heat maps, charts, and infographics to illustrate the distribution of evidence and gaps.

The dissemination of findings will be targeted towards a wide audience, including clinicians, researchers, decision-makers, and funding agencies. The goals will be to (a) guide researchers and funding agencies, highlighting priority areas for future research and funding to maximize the impact of AI on older adults’ health and safety through physical activity, and (b) bridge healthcare gaps, empowering healthcare providers to address existing gaps in services and improve overall health outcomes for older adults through exercise interventions.

### 5.2. Patient and Public Involvement

The development of the research question and outcome measures has incorporated input from patients. Patient representatives have been involved in the protocol and will continue to be engaged throughout the study. Their insights will help ensure that the research addresses their needs and experiences, particularly concerning the integration of AI technology in healthcare. Patient representatives will provide feedback on the study design, assist in defining relevant outcome measures, and participate in discussions about findings and recommendations, ensuring that their voices are integrated into the study’s outcomes.

## 6. Conclusions

This study will aim to provide a comprehensive understanding of the role of artificial intelligence in enhancing physical activity and mobility for older adults. A detailed evidence and gap map will visually highlight the strengths, gaps, and opportunities in the current evidence base. The findings will offer valuable data for researchers, clinicians, and policymakers, guiding future studies and funding priorities while empowering healthcare providers to leverage AI for improved health outcomes. The inclusion of patient perspectives will ensure that the study remains relevant and addresses real-world needs, fostering meaningful advancements in the integration of AI within healthcare for older adults.

## Figures and Tables

**Table 1:** Intervention (I) criteria for EGM AI-powered tools to enhance mobility and function in older adults.

Category	Type of Intervention	Description	Examples
Exercise and Rehabilitation Programs	AI-powered exercise and rehabilitation web applications	Use AI to personalize exercise routines based on user data including physical abilities and progress machine learning models (e.g., decision trees, neural networks, reinforcement learning).	Personalized workout plans: Custom exercise plans that adapt over time. Virtual coaching: Real-time guidance and feedback during exercises.
Functional Assessment Tools	AI tools for functional assessments and progress tracking	Assess physical function and monitors progress through video analysis or sensor data (computer vision algorithms such as Convolutional Neural Networks, CNNs) used for movement analysis or specific sensor fusion techniques used to gather and process data from wearables.	Movement analysis: Analyzes video recordings to assess technique. Wearable sensor integration: Tracks physical activity using wearable sensors.
Gamification and Motivation Tools	AI-powered gamification tools	Enhance engagement and motivation through game-like elements (adaptive learning algorithms that adjust the difficulty level based on a user’s performance or reinforcement learning to optimize user engagement).	Interactive games: Games requiring physical activity with performance feedback Rewards and challenges: Personalized challenges and rewards to boost participation.
Educational and Guidance Platforms	AI-driven educational platforms	Provide guidance on exercise techniques and health education using AI.	Instructional videos: Curated videos based on exercise history and goals. Health tips and alerts: Personalized tips and alerts related to physical activity.
Social Interaction and Support Networks	AI-powered social interaction platforms	Facilitate social interaction and peer support using AI (using Natural Language Processing (NLP) techniques, such as text classification or sentiment analysis, to ensure appropriate content moderation or the use of recommendation algorithms for connecting users with similar interests).	Social networks: Connecting users with peers for shared routines and support. Community forums: Moderated forums for sharing experiences and advice.

**Table 2:** Comparator (C) criteria for EGM AI-powered tools to enhance mobility and function in older adults.

Comparator	Description	Objective
Traditional exercise programs	Standard exercise regimens provided through printed materials, general exercise apps, or community classes.	To assess whether AI-powered interventions offer superior customization that allows for greater adherence or outcomes compared to traditional methods.
Non-AI-based digital tools	Digital applications that offer exercise routines but do not utilize AI features.	To evaluate whether AI-enhanced features result in better outcomes compared to other non-AI digital solutions.
Fitness coaching or Rehabilitation	Personalized coaching or rehabilitation by physical therapists or trainers without AI assistance.	To compare the effectiveness of AI tools with professional manual coaching and determine whether AI provides additional benefits.
No intervention (Control group)/waitlist	Participants who do not receive any structured exercise intervention.	To establish a baseline and measure the impact of AI-powered interventions against a group with no structured exercise.
Usual care	Standard care provided in the study setting including routine physical activity recommendations or rehabilitation services.	To compare AI-powered interventions adjuvant to routine care practices and assess the added value of AI tools.

**Table 3:** Outcome (O) criteria for EGM AI-powered tools to enhance mobility and function in older adults.

Outcome	Category	Description	Objective
Physical function and mobility	Muscle Strength and Endurance	Measurement of muscle strength and endurance through standardized tests.	Assess improvements in muscle strength and endurance due to AI-powered exercise interventions.
	Flexibility and Range of Motion (ROM)	Evaluation of joint flexibility and ROM using goniometers or other assessment tools.	Determine the effectiveness of AI interventions in enhancing flexibility and joint mobility.
	Balance and Stability	Assessment of balance and stability through tests such as the Berg Balance Scale or Timed Up and Go (TUG) test.	Evaluate the impact of AI applications on improving balance and reducing the risk of falls.
	Gait and Walking Speed	Measurement of gait parameters and walking speed using tools like wearable sensors or the 6-Minute Walk Test.	Analyze improvements in walking ability and mobility due to AI-powered interventions.
	Functional Fitness	Assessment of overall functional fitness through comprehensive tests such as the Senior Fitness Test.	Measure the overall improvement in physical function and fitness levels.
Patient reported outcome	Pain and Discomfort	Evaluation of pain levels using standardized pain scales like the Visual Analog Scale (VAS) or Numeric Rating Scale (NRS).	Assess the effectiveness of AI interventions in reducing pain and discomfort associated with physical activity.
	Quality of Life	Measurement of quality of life using validated questionnaires such as the SF-36 or EQ-5D.	Determine the broader impact of AI-powered exercise programs on overall quality of life and well-being.
Clinical/Safety outcomes	Fall	Falls are accidental events where a person unexpectedly ends up on the ground or a lower surface. They are especially common in older adults and those with balance or mobility challenges, often resulting in injuries, loss of independence, and higher healthcare needs [22]	Identify and describe AI-power-based exercise programs for older adults that aim to reduce fall risks. Highlight primary evidence gaps in AI interventions related to fall prevention and improving functional mobility. Evaluate the quality of existing reviews on the effectiveness of AI in preventing falls among older adults.
	Fracture	A fracture is a break in the structural integrity of the bone’s cortex, often accompanied by damage to the surrounding soft tissues [23]	Identify and describe AI-powered exercise programs for older adults that aim to reduce fracture risks. Highlight primary evidence gaps in AI interventions related to fracture prevention and improving functional mobility. Evaluate the quality of existing reviews on the effectiveness of AI in reducing fracture rates among older adults.
	Mortality	Number of deaths caused by the health event under investigation [24]	Identify and describe AI-power-based exercise programs for older adults aimed at improving mobility and reducing mortality risks or prevent mortality.
	Adverse events	An adverse event refers to any abnormal clinical finding linked to the use of a therapy. These events are classified based on their seriousness, expectedness, and relationship to the therapy [25]	Identify existing studies on the effectiveness of AI in both reducing adverse events in older adults engaged in exercise program or causing adverse events.
Engagement and usability	User Adherence and Engagement	Tracking user adherence to the exercise program and their engagement levels using usage data and self-reports.	Analyze the acceptability and sustainability of AI-powered interventions among older adults.
	User Satisfaction and Experience	Measurement of user satisfaction and experience through surveys and feedback forms.	Gather data into the user-friendliness and perceived value of the AI applications.
	Technology Usability	Evaluation of the usability of the AI-powered system using standardized usability scales such as the System Usability Scale (SUS).	Identify potential barriers to usage and areas for improvement in the design.

## Data Availability

The data sets generated during and/or analyzed during this study will be available from the corresponding author on reasonable request.

## Appendix A. Literature Search MEDLINE

1 artificial intelligence/
2 ((artificial adj3 intelligen*) or (computation* adj3 intelligen*) or (machine* adj3 intelligen*) or (computer* adj3 reason*) or computer vision system* or (knowledge adj3 represent*) or (knowledge adj3 acquisition)).tw,kf.
3 (AI or AIVI or genAi).tw,kf.
4 exp Machine Learning/
5 ((deep adj3 learn*) or (hierarchical adj3 learn*) or (machine adj3 learn*) or (transfer adj3 learn*)).tw,kf.
6 (opinion mining or (sentiment adj3 analy*) or (sentiment adj3 classif*) or support vector machine*).tw,kf.
7 (Runway AI or Runway Gen-1 or “Bing chat” or ChatGPT* or “Chat GPT” or “Dall-E” or DallE or “DeepAI Google* Bard” or “Google* Gemini” or “IBM Watson” or InVideo or “Microsoft* Bing” or “Microsoft* Copilot” or Midjourney or OpenAI or “Open AI” or PathAI or “Path AI” or Perplexity).mp.
8 (“classification algorithm*” or “computer heuristic*” or “convolutional network*” or “decision support system*” or “decision tree” or “data science” or “feature detection” or “generative pre-trained transformer” or “generative pretrained transformer” or “language learning model*” or “large language model*” or “learning algorithm*” or (Markov adj3 model*) or ((multifactor* or multicriteria) adj3 (“decision analysis” or “decision making”)) or “natural language process*” or “nearest neighbo*” or “neural network*” or “outlier detection” or “pattern recognition” or “probability tree” or “random forest” or “representation learning”).tw,kf.
9 or/1–8 [Artificial Intelligence]
10 exp Exercise/
11 exp Exercise Therapy/
12 physical therapy modalities/or exp exercise movement techniques/or exp post-exercise recovery techniques/
13 exp Physical Fitness/
14 exp Postural Balance/
15 exp Sports/
16 movement/or locomotion/
17 exercis*.tw,kf./freq=2 or exercis*.ti.
18 (athlete* or athletic* or (balanc* adj1 exercis*) or coach* or enduran* or fitness or movement analys* or mobility or movement therap* or plyometric* or postur* or propriocep* or resist* train* or restric* train* or strength train* or stretch* or weight train* or lift weigh* or workout* or “working out”).tw,kf.
19 (aerobic* or aikido or badminton or baseball or basketball or bicyl* or biking or bouldering* or bowler* or bowling or boxing or boxer* or (chair adj1 exercis*) or climbing or climber* or cricket or cycling* or cyclist or dance or dancer* or dancing or diving or diver or divers or diving or football or golf* or gong fu or gongfu or gymnast* or hiker* or hiking* or hockey* or jogging or jogger* or Jujitsu or judo or karate or kung fu or lacrosse or martial art* or mountaineering or netball or pickleball or pickle ball or racquet* or racket* or rowing or rower* or rugby or runner* or skater* or skating or skateboard* or skate board* or ski or skiing or skier* or snowboard* or snow board* or soccer* or softball or sport* or squash or swimming or swimmer* or surfing or surfer* or tai chi or tai ji or tennis or volleyball or walking or water polo or wrestler or wrestling or yoga).tw,kf.
20 or/10–19 [Exercise or Movement]
21 exp aged/or exp geriatrics/or exp geriatric nursing/or (centarian* or centenarian* or elder* or eldest or frail* or geriatri* or nonagenarian* or octagenarian* or octogenarian* or old age* or older adult* or older age* or older female* or older male* or older man or older men or older patient* or older people or older person* or older population or older subject* or older woman or older women or oldest old* or senior* or senium or septuagenarian* or supercentenarian* or very old*).ti,ab,kf. [Elderly Concept]
22 9 and 20 and 21

## Appendix B. AMSTAR-2 Checklist for Analyzing Systematic Reviews on AI Interventions for Improving Mobility and Function in Older Adults

Reference for the adapted tool: Veras, M., Pardo, J., Lê, M., Jussup, C., Tatmatsu-Rocha, J. C., & Welch, V. (2024). AMSTAR-2 Checklist for Analyzing Systematic Reviews on AI Interventions for improving mobility and function in Older Adults. in Protocol for AI-powered tools to enhance mobility and function in older adults—An evidence and gap map. Journal of Personalized Medicine, jpm-15-00029. Adapted from: Shea, B. J., Reeves, B. C., Wells, G., Thuku, M., Hamel, C., Moran, J., Moher, D., Tugwell, P., Welch, V., Kristjansson, E., & Henry, D. A. (2017). AMSTAR 2: A critical appraisal tool for systematic reviews that include randomised or non-randomised studies of healthcare interventions, or both. BMJ, 358, j4008. https://doi.org/10.1136/bmj.j4008 [29].

**1. Did the research questions and inclusion criteria for the review include the components of PICO (Population, Intervention, Comparator, Outcome)?**

Yes: The systematic review explicitly defines research questions and inclusion criteria that clearly include the components of PICO, with a focus on older adults, AI-powered interventions (e.g., wearable devices, rehabilitation tools), appropriate comparators (e.g., traditional methods), and mobility/function outcomes.

Partial Yes: The review includes PICO components but does not clearly specify all elements (e.g., missing details about comparators or outcomes).

No: The review does not adequately define the PICO components in relation to AI interventions for older adults.

**2. Did the report of the review contain an explicit statement that the review methods were established prior to the conduct of the review and did the report justify any significant deviations from the protocol?**

Yes: In addition to meeting all the criteria for Partial Yes, the protocol should also have been registered and specified additional elements such as a meta-analysis or synthesis plan (if appropriate) and a more detailed plan for investigating causes of heterogeneity.

Partial Yes: the authors stated that they had a written protocol or guide that included all the following: review question(s), a search strategy, inclusion/exclusion criteria, a plan for investigating causes of heterogeneity, and a risk of bias assessment.

No.

**3. Did the review authors explain their selection of the study designs for inclusion in the review (e.g., RCTs, observational studies)?**

Yes: The review provides a clear explanation for the inclusion of study designs assessing AI-powered interventions for older adults, including randomized controlled trials (RCTs) and non-randomized studies of interventions (NRSIs) when applicable. It explicitly justifies why both types of studies are appropriate for evaluating the effectiveness of AI interventions for improving mobility and function in older adults.

No: The review does not adequately explain or justify the inclusion of specific study designs (e.g., RCTs or NRSIs) in the context of AI interventions for older adults.

OR Explanation for including only RCTs: The review includes only randomized controlled trials (RCTs) for evaluating AI interventions for older adults, but does not clarify why NRSIs were excluded.

OR Explanation for including only NRSIs: The review includes only non-randomized studies of interventions (NRSIs) in its analysis of AI-powered interventions for older adults, without justifying the exclusion of RCTs.

OR Explanation for including both RCTs and NRSIs: The review justifies the inclusion of both RCTs and non-randomized studies of interventions (NRSIs) to assess AI interventions for older adults, with appropriate reasoning for why both study designs are valuable for understanding the effectiveness of AI tools on functional improvement and mobility.

**4. Did the review authors use a comprehensive literature search strategy?**

Partially Yes: The authors searched at least two databases relevant to the research question, provided keywords and/or a search strategy, and justified publication restrictions (e.g., language). However, they did not meet all the additional criteria, such as searching reference lists or bibliographies, consulting content experts, or conducting the search within 24 months of completing the review.

Yes: The authors searched at least two databases relevant to the research question, provided keywords and/or a search strategy, justified publication restrictions (e.g., language), and also searched the reference lists or bibliographies of included studies, searched trial/study registries, consulted content experts in the field, searched for grey literature where relevant, and conducted the search within 24 months of completing the review.

No: The authors did not meet the basic criteria for Partial Yes, such as searching at least two relevant databases, providing keywords or a search strategy, or justifying publication restrictions. Additionally, they did not fulfill the additional requirements for Yes, such as searching reference lists, consulting content experts, searching for grey literature, or conducting the search within 24 months of completing the review.

**5. Did the review authors perform study selection in duplicate?**

Yes: either of the following criteria must be met: at least two reviewers independently agreed on the selection of eligible studies and achieved consensus on which studies to include, or two reviewers selected a sample of eligible studies and achieved good agreement (at least 80 percent), with the remainder selected by one reviewer.

No: the authors did not meet either of the above criteria. This means that the selection of eligible studies was not done independently by two reviewers, or there was insufficient agreement between reviewers on which studies to include.

**6. Did the review authors perform data extraction in duplicate?**

Yes: either of the following criteria must be met: at least two reviewers achieved consensus on which data to extract from included studies, or two reviewers extracted data from a sample of eligible studies and achieved good agreement (at least 80 percent), with the remainder extracted by one reviewer.

No: the authors did not meet either of the above criteria. This means that data extraction was not done by at least two reviewers who reached consensus, or there was insufficient agreement between reviewers on the data to be extracted from the included studies.

**7. Did the review authors provide a list of excluded studies and justify the exclusions?**

Yes: The review provides a list of all studies considered but excluded, with clear justifications for exclusion, especially regarding AI interventions for older adults.

Partial Yes: The review lists excluded studies, but the justifications for exclusions are not entirely clear or are lacking details about AI interventions.

No: No list or justification for excluded studies is provided.

**8. Did the review authors describe the included studies in adequate detail?**

Yes: The review describes the included studies in detail, including the population (older adults), AI interventions (e.g., wearable devices, rehabilitation tools), outcomes (mobility, function), and study design.

Partial Yes: The review provides some details about the studies, but certain aspects (e.g., AI interventions or outcomes) are not sufficiently described.

No: The review lacks sufficient detail about the studies, particularly regarding the AI interventions or outcomes.

**9. Did the review authors use a satisfactory technique for assessing the risk of bias (RoB) in individual studies that were included in the review?**

Yes: the review comprehensively assessed the risk of bias (RoB) across all key domains for both randomized controlled trials (RCTs) and non-randomized studies of intervention (NRSIs). This included addressing specific considerations for AI interventions, such as algorithmic bias and data transparency. Additionally, the review assessed key domains such as unconcealed allocation, allocation sequence, blinding of patients and assessors (when necessary), methods used to ascertain exposures and outcomes, and the selection of the reported result from among multiple measurements or analyses of a specified outcome.

Partial Yes: the review assessed some important RoB domains, but it did not fully address all critical aspects, particularly those specific to AI interventions. For example, it may have assessed some of the key domains like confounding or selection bias but lacked comprehensive evaluation of other important factors such as algorithmic bias or data transparency, or it did not fully address issues related to selection of the reported result or lack of blinding in certain cases.

No: the review did not adequately assess the risk of bias or omitted key domains relevant to AI-based studies. It may have failed to address critical areas like allocation sequence, selection bias, confounding, or the reporting of results, and did not include specific considerations for AI interventions such as algorithmic bias or data transparency.

**10. Did the review authors report on the sources of funding for the studies included in the review?**

Yes: The review reports on the sources of funding for the included studies, including those that evaluated AI interventions for older adults.

Partial Yes: Some sources of funding are reported, but it is not clear whether funding for AI-related studies is disclosed.

No: No information on funding sources is provided.

**11. If meta-analysis was performed, did the review authors use appropriate methods for statistical combination of results?**

Yes: If meta-analysis was conducted, appropriate methods (e.g., random-effects models or other statistical models) were used to combine results from studies on AI interventions. The analysis effectively accounted for heterogeneity in AI-based interventions, such as the use of machine learning models (e.g., decision trees, neural networks, reinforcement learning) for personalizing exercise routines, or the application of computer vision algorithms (e.g., Convolutional Neural Networks—CNNs) and sensor fusion techniques for analyzing movement and monitoring progress. This ensures that variations in AI technologies and their application to different populations (e.g., older adults) are adequately considered in the statistical synthesis.

Partial Yes: Meta-analysis was performed, but the methods used to combine results from AI intervention studies (e.g., random-effects models) or the treatment of heterogeneity (e.g., considering the different AI technologies like machine learning and computer vision algorithms) were not fully adequate. The review might have overlooked some sources of variation, such as differences in AI models or the diversity of outcomes measured (e.g., mobility, physical function, user engagement).

No: No meta-analysis was performed, or the methods used to combine results from AI intervention studies were not appropriate for handling the unique aspects of AI-based interventions. For example, the analysis did not account for the complexity of AI algorithms (like sensor fusion or machine learning models) or failed to address heterogeneity arising from the use of different AI technologies and their varying impacts on older adults’ mobility and functional improvement.

**12. If meta-analysis was performed, did the review authors assess the potential impact of RoB in individual studies on the results of the meta-analysis or other evidence synthesis?**

Yes: The review investigates how the risk of bias in studies evaluating AI interventions for older adults may impact the meta-analysis results.

Partial Yes: The review mentions potential bias but does not fully assess or account for its impact on meta-analysis results.

No: The review does not address the potential impact of risk of bias on the meta-analysis results.

**13. Did the review authors account for RoB in individual studies when interpreting/discussing the results of the review?**

Yes: The review discusses how biases in individual studies (e.g., AI intervention studies) may have affected the results and interpretation of the findings. This includes addressing potential biases unique to AI studies, such as the limitations in AI model training (e.g., biases in the data used to train machine learning models), inaccuracies in sensor data collection (e.g., from wearables or movement analysis using computer vision algorithms), or issues related to the application of AI tools in diverse populations (e.g., older adults with varying physical capabilities). The review also discusses how these biases could affect the generalizability of the findings, especially considering the use of personalized AI technologies.

No: The review does not address or discuss the impact of risk of bias on the results. This includes failing to consider how biases specific to AI interventions (e.g., overfitting in machine learning models, biases in sensor data from wearable devices) may have influenced the study outcomes, or neglecting to discuss how these biases could limit the reliability or generalizability of the results.

**14. Did the review authors provide a satisfactory explanation for, and discussion of, any heterogeneity observed in the results of the review?**

Yes: The review provides a clear explanation of heterogeneity in the results, exploring differences across studies of AI interventions for older adults. It investigates potential sources of heterogeneity specific to AI-based interventions, such as variations in the types of AI tools used (e.g., machine learning models, sensor technologies, or computer vision algorithms), differences in study populations (e.g., varying levels of mobility, frailty, or health status in older adults), and variations in the implementation of AI interventions (e.g., AI-powered exercise programs versus rehabilitation tools). The review may also discuss how factors like AI model customization, data collection methods (e.g., from wearable devices or video analysis), and the level of personalization in AI interventions (e.g., adapting to individual progress or needs) contribute to heterogeneity in the results.

No: The review does not discuss heterogeneity or investigate its sources. There is no mention of differences in study results, nor an attempt to understand what might have caused those differences, especially with respect to the use of AI interventions for older adults.

**15. If they performed quantitative synthesis, did the review authors carry out an adequate investigation of publication bias (small study bias) and discuss its likely impact on the results of the review?**

Yes: The review employed appropriate statistical tests (e.g., funnel plots or Egger’s test) to assess publication bias related to studies on AI interventions for older adults. It discussed the potential impact of publication bias, particularly how the results of smaller studies on AI-powered interventions (such as wearable devices or rehabilitation tools) might be skewed or overrepresented in the literature. The review may have explored how negative or null results from smaller studies could be underreported or excluded, and the potential implications for generalizing the findings of AI interventions across broader populations of older adults.

No: No investigation of publication bias was conducted. The review did not use statistical methods to assess publication bias or failed to discuss the impact of potential bias, leaving unanswered questions about how smaller studies on AI interventions for older adults might influence the overall conclusions.

No Meta-analysis conducted

**16. Did the review authors report any potential sources of conflict of interest, including any funding they received for conducting the review, particularly related to AI interventions?**

Yes: The authors reported no competing interests, and they clearly described any funding sources, particularly those related to AI interventions for older adults (e.g., funding from companies developing wearable devices or AI-based rehabilitation tools). They also explained how they managed potential conflicts of interest, such as disclosures of funding from AI technology companies or stakeholders involved in the development of the interventions studied. This transparency ensures that the review’s findings are not biased by financial or personal interests linked to AI technologies or products.

No: The authors did not report any potential sources of conflict of interest or funding, or they failed to disclose relevant financial ties to organizations or companies involved in AI interventions for older adults. This lack of transparency raises concerns about the potential influence of financial interests on the study’s findings and conclusions regarding AI-powered interventions.
